# Caring for a retinal camera

**Published:** 2023-07-07

**Authors:** Ismael Cordero

**Affiliations:** 1 Clinical Engineer, Philadelphia, USA.

**Maintenance and care will prolong the life of this expensive item.**

Retinal cameras, also known as fundus cameras, are used to take pictures of the back of the eye.

A retinal camera consists of a specialised microscope with an integrated or attached camera. There are two main types: table top (Figure 1) and hand held.

**Figure 1:**
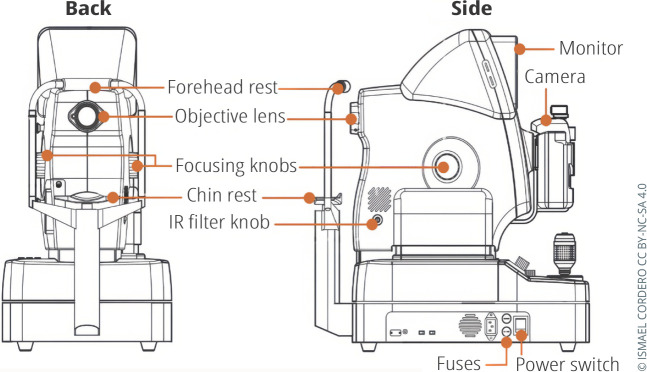
Diagram of a table-top retinal camera.

**Important**

Before you start any cleaning, disinfection, or maintenance activities, consult the instructions for the specific model of retinal camera. The use of improper cleaning agents and procedures may void the warranty and degrade the equipment.

## Daily care

**At the start of each screening session**, check the objective lens to make sure it is free of dirt. Clean if needed (see panel).

**After each patient**, clean and disinfect the parts of the camera that were in contact with the patient, mainly the forehead rest and chin rest (see panel). This reduces the risk of spreading infections between patients.

### At the end of each screening session

- Turn off the instrument
- Clean the camera body (see panel) and objective lens
- Cover the objective lens with the protective lens cap
- Cover the instrument with its dust cover. **Note:** never replace the dust cover when the illumination is on.

## Storage

If you intend not to use the camera for a long period of time, take the plug out of the socket and replace the lens cap and dust cover. Keep the instrument in a dry and well-ventilated area; this will help to prevent fungal growth.

## Maintenance tips

- When replacing bulbs, do not touch them with your bare hands. Touching bulbs may shorten their life and reduce the amount of light they emit.
- When replacing any fuses, make sure that the instrument is turned off and unplugged. Wait at least 5 minutes for the power supply to discharge.
- Have the instrument checked and maintained regularly by the vendor at least once every two years to confirm its performance and safety. Consult your dealer for details and cost of the inspection.

**General instructions for cleaning**

**Note:** these instructions are generic and may not be applicable to all models of retinal cameras. Always consult the instructions for the specific model of retinal camera you are using.

**Clean and disinfect the forehead and chin rests**

Lightly dampen a cloth with ethyl alcohol solution (75% maximum), then wipe the forehead rest and chin rest, and let them air dry. If ethyl alcohol is not available, use a solution of 0.05% sodium hypochlorite; however, this may cause faster deterioration of the materials.

**Clean the camera body**

1. Wipe dust off the camera body using a dry, soft cloth.
2. Use a soft cloth lightly dampened with ethyl alcohol solution (75% maximum) to disinfect the camera body and remove stains.
3. Let the camera body air dry.

**Note:** Be careful not to get moisture inside the camera body. Never use solvents or other abrasive agents.

**Clean the lens**

**Figure 2:**
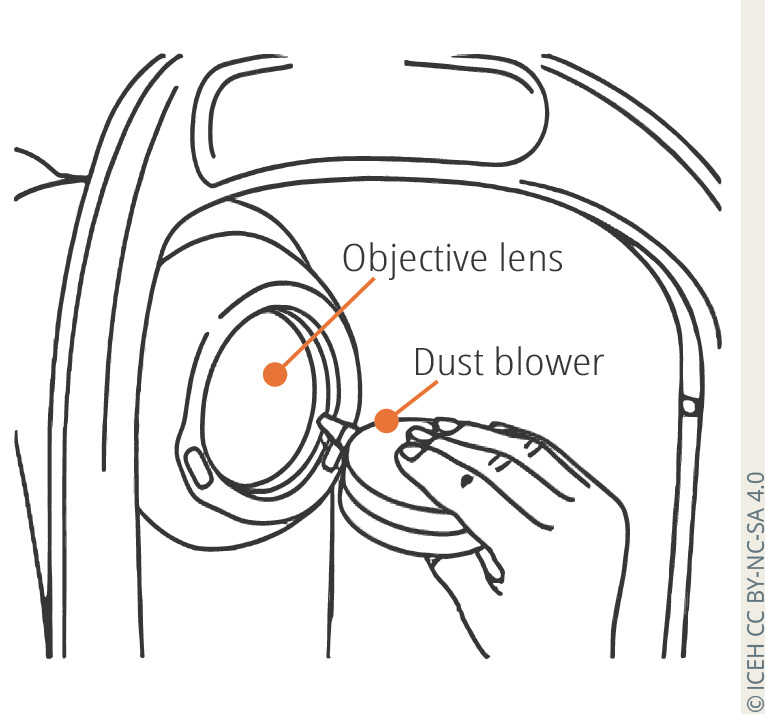
Using a dust blower to clean the lens.

1. Turn on the power.
2. Darken the room and then adjust the infrared (IR) filter knob to change the illumination light to visible radiation. This makes it easier to see dirt on the lens.
3. Set the light intensity control knob to its maximum.
4. Blow off any dust on the objective lens using a dust blower (Figure 2).
5. If the dust blower is inefficient, wipe the surface using lens cleaning paper moistened with a manufacturer-approved lens-cleaning solution. Be sure to wipe carefully and gently, without applying force. Rotate the wipe little by little in a circular motion, working from the centre of the lens toward the edge.

If optical lens cleaning paper is not available in your area, you may instead use a lint-free, non-abrasive cloth.

If optical cleaning solution is not available, try the following solutions, from weakest (1) to strongest (4), until the surface is clean:

1. Distilled water
2. A solution of 1 part mild, pH-neutral detergent to 19 parts distilled water
3. A mixture of 60% acetone and 40% methanol (not for use on plastic lenses)
4. Isopropyl alcohol (90% solution). **Note:** slow evaporation can leave drying marks on the surface.

